# Electrocorticography to Investigate Age-Related Brain Lateralization on Pediatric Motor Inhibition

**DOI:** 10.3389/fneur.2022.747053

**Published:** 2022-03-07

**Authors:** Chao-Hung Kuo, Kaitlyn Casimo, Jing Wu, Kelly Collins, Patrick Rice, Bo-Wei Chen, Shih-Hung Yang, Yu-Chun Lo, Edward J. Novotny, Kurt E. Weaver, You-Yin Chen, Jeffrey G. Ojemann

**Affiliations:** ^1^Department of Biomedical Engineering, National Yang Ming Chiao Tung University, Taipei, Taiwan; ^2^School of Medicine, National Yang Ming Chiao Tung University, Taipei, Taiwan; ^3^Department of Neurosurgery, Neurological Institute, Taipei Veterans General Hospital, Taipei, Taiwan; ^4^Department of Neurological Surgery, University of Washington, Seattle, WA, United States; ^5^Graduate Program in Neuroscience, Center for Neurotechnology, University of Washington, Seattle, WA, United States; ^6^Department of Bioengineering, Center for Neurotechnology, University of Washington, Seattle, WA, United States; ^7^Department of Neurological Surgery, Oregon Health & Science University, Portland, OR, United States; ^8^Department of Psychology, Institute for Learning and Brain Sciences, University of Washington, Seattle, WA, United States; ^9^Department of Mechanical Engineering, National Cheng Kung University, Tainan, Taiwan; ^10^The Ph.D. Program for Neural Regenerative Medicine, College of Medical Science and Technology, Taipei Medical University, Taipei, Taiwan; ^11^Departments of Neurology and Pediatrics, University of Washington, Seattle, WA, United States; ^12^Center for Integrative Brain Research, Seattle Children's Research Institute, Seattle, WA, United States; ^13^Department of Radiology, Integrated Brain Imaging Center, University of Washington, Seattle, WA, United States; ^14^Center for Neurotechnology, University of Washington, Seattle, WA, United States; ^15^Departments of Surgery, Seattle Children's Hospital, Seattle, WA, United States

**Keywords:** electrocorticography, Go/No-Go, high-gamma, inferior frontal gyrus, lateralization, motor inhibition

## Abstract

Response inhibition refers to the ability to suppress inappropriate actions that interfere with goal-driven behavior. The inferior frontal gyrus (IFG) is known to be associated with inhibition of a motor response by assuming executive control over motor cortex outputs. This study aimed to evaluate the pediatric development of response inhibition through subdural electrocorticography (ECoG) recording. Subdural ECoG recorded neural activities simultaneously during a Go/No-Go task, which was optimized for children. Different frequency power [theta: 4–8 Hz; beta: 12–40 Hz; high-gamma (HG): 70–200 Hz] was estimated within the IFG and motor cortex. Age-related analysis was computed by each bandpass power ratio between Go and No-Go conditions, and phase-amplitude coupling (PAC) over IFG by using the modulating index metric in two conditions. For all the eight pediatric patients, HG power was more activated in No-Go trials than in Go trials, in either right- or left-side IFG when available. In the IFG region, the power over theta and HG in No-Go conditions was higher than those in Go conditions, with significance over the right side (*p* < 0.05). The age-related lateralization from both sides to the right side was observed from the ratio of HG power and PAC value between the No-Go and Go trials. In the pediatric population, the role of motor inhibition was observed in both IFG, with age-related lateralization to the right side, which was proved in the previous functional magnetic resonance imaging studies. In this study, the evidence correlation of age and response inhibition was observed directly by the evidence of cortical recordings.

## Introduction

Motor inhibition refers to the ability to suppress inappropriate or prepotent actions that interfere with goal-driven behavior. Go/No-Go tasks are designed to provide experimental epochs of movement preparation, response execution, and motor inhibition. Consequently, they are widely used to investigate neural responses specifically attributable to motor inhibition (1). Typically, during the task, participants are requested to press a button or otherwise respond to one type of stimuli, such as a set of alphabet letters, a colored dot, or an image (Go stimuli), and withhold or inhibit a response to another type of stimuli, such as a specific single letter, a different color dot, or a contrasting image (No-Go stimuli). Inhibition is a conversion process of motor behavior, reflecting the capacity to selectively withhold voluntary movements (2).

A large accumulation of neuroscientific evidence from functional (fMRI) studies has reported an increase in the concentration of oxygenated hemoglobin during successful No-Go inhibition in the predominantly right-lateralized brain network comprising the orbitofrontal cortex, dorsolateral pre-frontal cortex, supplementary motor areas (SMA)/pre-SMA areas, basal ganglia circuits, and inferior frontal gyrus (IFG) (3–6). The results indicate that motor inhibition is a largely lateralized process within (generally) the right hemisphere, with the right IFG believed to be particularly sensitive to response suppression (7). Within this inhibition network, the right IFG is predicted to serve as an execution center when inhibition is required (8–10).

Following a literature review, a power spectrum analysis based on adults' electroencephalogram (EEG) studies demonstrated that the theta frequency band (4–8 Hz) plays an important role during motor inhibitory control (11–13). Considering the demand for motor inhibition, theta band activity revealed a correlation with right IFG during inhibition tasks (14). The power change between the Go and No-Go conditions were also observed in electrocorticography (ECoG) studies. A previous ECoG study of 16 patients with intracranial electrodes recording for medically refractory epilepsy found significantly increased gamma band activity in the right IFG after No-Go signal cueing (15). Motor inhibition-evoked gamma-band responses during No-Go trials localize to the right IFG (15), which is compatible with the activation of blood oxygenation level-dependent (BOLD) response in functional MRI (fMRI) studies (3, 8). The aforementioned findings, based on EEG and ECoG studies, show that the right IFG has a role in the motor inhibition of adults.

Age-related functional and anatomical neural development has been observed (16). In a meta-study including 2–12-year-old children from 65 EEG studies, the No-Go-related negative amplitude became progressively negative by age, compared with the Go conditions. The results implied that No-Go-related EEG signals as indexing motor inhibition would change with age (17). In the pediatric population, the findings of frontal activation in No-Go conditions were similar to those in adult populations (2, 18, 19), with some notable differences, including an overall greater engagement of more widespread brain networks (19) and left frontal engagement, which was predicted to contribute to motor inhibition in the immature nervous system (20). Yet, to date, the vast majority of electrophysiological investigations of response in inhibition have been based on adult populations; the dominance of right IFG, the contributions of left IFG during motor inhibition, and the connectivity of frontal and other parts of brain regions between Go and No-Go conditions in children have not been explored using ECoG. Because subdural recordings provide the greatest fidelity of gamma-band dynamics, confirmatory recordings using ECoG in pediatric populations during response-inhibition are warranted.

The processing of motor inhibition included not only IFG, but also other parts of the brain, including the pre-SMA, orbitofrontal cortex, dorsolateral pre-frontal cortex, and basal ganglia (3–6). Considering the neural network of motor inhibition, power changes across different parts of the cortex between Go and No-Go conditions were proven to be related to cortical connectivity and response accuracy in tasks (11, 21). Thus, taken all together, phase-amplitude coupling (PAC) is a suitable cross-frequency coupling approach used to evaluate cortical coupling and functional connectivity. For example, in a magnetoencephalography (MEG) study, high-gamma power (HG, 30–70 Hz) was phase-locked to alpha neural oscillation (8–13 Hz) with the human eyes closed within occipital channels (22). Furthermore, in ECoG studies, the phase of canonical low-frequency bands, such as theta (4–8 Hz), has been shown to modulate power in HG band (80–150 Hz) signals (23). The task-related coupling effects were also observed between the phase of low-frequency (0–3.5 Hz) and amplitude of gamma band (28–70 Hz) neural signals (24). Comparing the coupling effect between the ECoG and fMRI signals in the resting state of brain connectivity, PAC mimicked comparable patterns in these two measurements (25). Collectively, it is believed that PAC represents a neural gating mechanism where the presence of significant coupling indicates the phase of a low-frequency oscillator, which serves to briefly facilitate the high-frequency activity of a second, distant cortical region (23, 26).

It has been predicted that PAC may serve as a neurophysiological mechanism underlying the maturation of neural communication (26) and as a driving mechanism for orchestrating function, including motor inhibition (27). However, the degree to which this neural network and physiological mechanism of motor inhibition exist within pediatric populations is unclear. The aim of this study is to investigate the neural activity, extracted from in-dwelling ECoG electrodes, during motor inhibition in the pediatric population, and estimate the degree to which PAC is associated with the maturation of motor inhibition.

## Materials and Methods

### Participants

A total of eight pediatric patients (male:female = 3:5; mean age 9.8 years; range 7–16) underwent neurological surgery at the Seattle Children's Hospital in Seattle, Washington, for the treatment of intractable epilepsy without evidence of anatomical abnormality from the MRI examinations. Corticographic potentials were acquired from four patients with right hemisphere grids, and four patients with left-sided grids, according to the clinical considerations. Five patients completed Go/No-Go testing for both the hands and the other three patients only performed right-hand testing as shown in Table 1. With approval from the Seattle Children's Hospital Institutional Review Board, all patients and guardians provided informed consent, including the use of ECoG recordings and medical records. All patients underwent a two-stage surgery: craniotomy with unilateral subdual grid and strip implantation according to seizure semiology, followed by removal of the electrodes with resection of epileptic foci. Subdural ECoG 8 × 8 grids or 2 × 8 strips (Integra, Princeton, New Jersey, USA) with 4.75-mm diameter platinum electrodes spaced at 10 mm were transiently placed subdurally to localize the epileptic foci according to clinical considerations, and removed after 1 week of ECoG monitoring.

**Table 1:** Demographical and clinical characteristics of pediatric patients implanted ECoG grids.

**Patient no**.	**Age**	**Gender**	**Grid location**	**Side**	**Handedness**	**Hand testing**	**Performance (accuracy, %)**
1	7	F	Frontal/Parietal	Right	Right	Right	64.2
2	8	F	Frontal/Parietal	Left	Right	Right	93.9
3	9	M	Frontal/Parietal/Temporal	Left	Right	Both	74.5
4	11	F	Frontal/Parietal/Temporal	Right	Right	Both	95.9
5	11	F	Frontal/Parietal/Temporal	Left	Right	Both	75.0
6	12	M	Frontal/Temporal	Right	Right	Both	92.9
7	15	F	Frontal/Parietal/Temporal	Right	Right	Both	66.3
8	16	M	Frontal/Parietal/Temporal	Left	Right	Right	97.9

### Go/No-Go Task and Signal Recording

**Figure 1:**
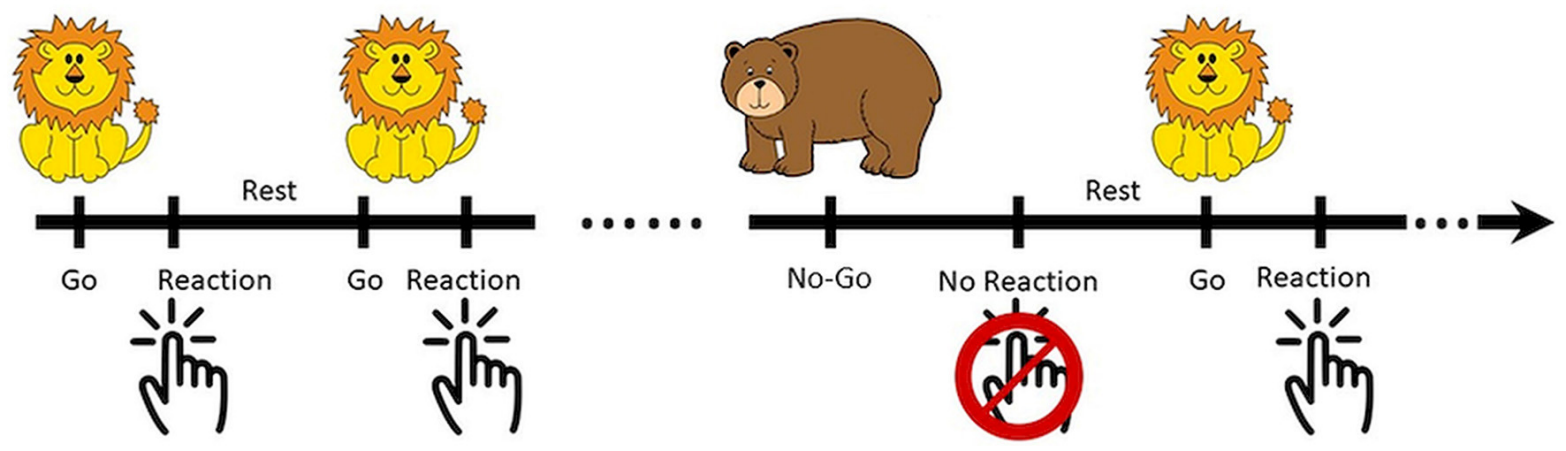
Illustration of the optimized Go/No-Go paradigm (Go vs. No-Go = 1:6) for children. The patients were asked to respond in Go trials (lion) and hold in No-Go trials (bear).

The Go/No-Go task was developed in Psychotoolbox-3 with MATLAB software (Mathworks, Natick, Massachusetts, USA) and was optimized for children (28–30). During the task, patients were asked to press a button on the appearance of a Go signal (a lion), and not respond on the appearance of a No-Go signal (a bear). The No-Go vs. Go signals were randomly distributed at the ratio of 1:6 with a total of 49 trials in each experimental run. There was a 1 s jittered intertrial interval as shown in Figure 1. Patients were asked to use the right hand during the first run of the task, and the left hand for the 2nd round. However, not all the patients complied. The responses were classified as Go-correct (lion, reaction), Go-wrong (lion, no reaction), No-Go-correct (bear, no reaction), and No-Go-wrong (bear, reaction).

The ECoG signals were recorded at 1.2 kHz by the clinical system with the Xltek® or Natus® Quantum® LTM amplifier (Natus, Pleasanton, California, USA). A portable laptop was utilized for task execution and documenting patients' responses, which were recorded simultaneously with ECoG signals. A digital transistor-transistor logic (TTL) output signal generated through Psychotoolbox-3 time-stamped event boundaries on the ECoG time series data.

### Electrocorticography Data Analysis

**Figure 2:**
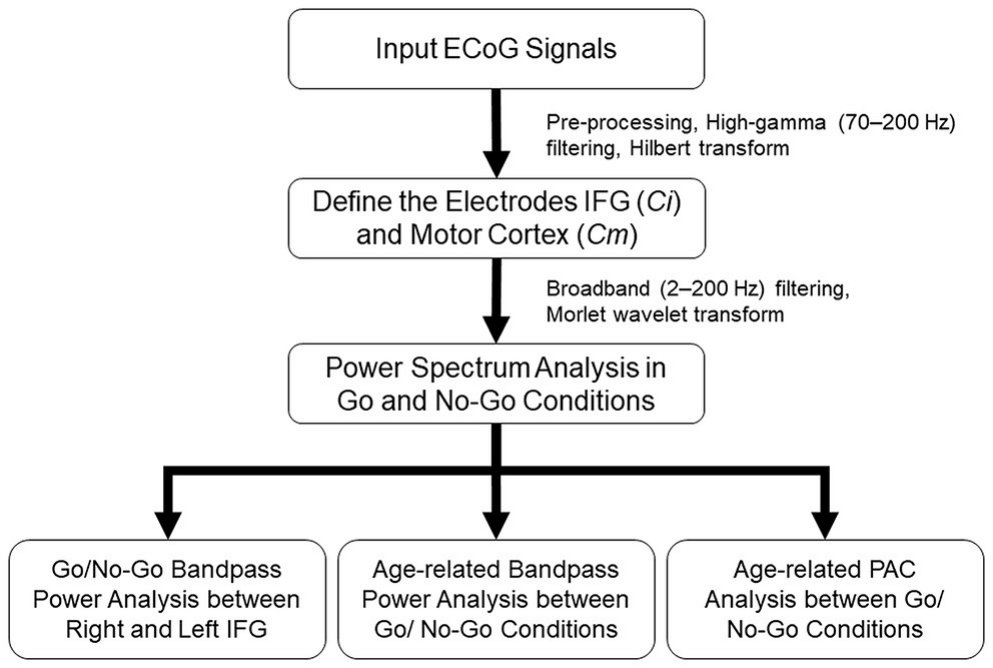
Illustration of the flow chart of data analysis. After preprocessing the ECoG signal, high-gamma (HG) activation was applied to define the most activated electrode to represent inferior frontal gyrus (IFG) (*Ci*) and motor cortex (*Cm*). Broadband pass power spectrum was analyzed between Go and No-Go conditions. Finally, side-related bandpass power changed, as well as age-related bandpass power and phase-amplitude coupling (PAC) between Go and No-Go conditions were further evaluated.

All the artifact-free ECoG signals were analyzed with MATLAB software (R12, MathWorks, Natick, Massachusetts, USA). The flowchart of ECoG preprocessing was shown in Figure 2. Data were rereferenced within the grid by common average and notch filtered for line noise (at 60, 120, and 180 Hz). A spectral density time series was computed for each channel by (HG, 70–200 Hz) bandpass filtering (4th-order zero-phase Butterworth filter) and the absolute values of the Hilbert transform of the filtered signals were estimated. The bandpass filtered time series were binned into response categories and their corresponding HG powers for each trial type within each epoch were calculated from 1 s before to 1 s after the visual cue. The time-series data was then z-normalized to the first second of (i.e., baseline, precue period of the trial) signal.

The coordinates of each electrode were recorded by the intra-operative navigation system and transformed into patient space, which was defined by the preoperative high-resolution T1-weighted MRI volume. The data was further transformed to MNI standard space for the identification of motor and motor areas according to anatomical structure (31). Based on the patients' responses, the results would be Go/Correct, Go/Non-correct, No-Go/Correct, and No-Go/Non-correct. As a screening and selection measure for electrodes over IFG and motor cortex, the maximum value of each trial within an epoch was averaged in Go/Correct and No-Go/Correct conditions. For visualization purposes only, *Z*-score values in the electrodes over the IFG and motor cortex were plotted on the MNI brain and spatially normalized by a Gaussian distribution. Heat (i.e., color) maps were created to reveal HG brain activity in Go/Correct and No-Go/Correct conditions. All the electrodes within the IFG and motor cortex were ranked according to epoch-based normalized-HG activity. For each patient, the most activated electrodes within Go/Correct and No-Go/Correct conditions were identified as motor cortex cortical signal (*Cm*) and IFG cortical signal (*Ci*), respectively, and selected for statistical and cross-frequency coupling analysis.

Broadband power spectra of only *Cm* and *Ci* were further calculated. Raw time series data from *Cm* and *Ci* were bandpass filtered (2–200 Hz, 4th-order zero-phase Butterworth filter); the Morlet wavelet transform was calculated on the bandpass filtered series (32–34) and truncated to each epoch of Go and No-Go conditions from 1 s before to 1 s after the visual cue. For each epoch, the calculations of phase and amplitude of the measured ECoG were determined through the Morlet wavelet transform. Amplitude estimates were again *Z*-normalized by the first second of power of each 3 Hz frequency step and averaged across all epochs of Go/Correct and No-Go/Correct conditions. The power change across low-to-high frequencies, from rest (1 s before the visual cue) to reaction (1 s after the visual cue), was estimated in Go/Correct and No-Go/Correct conditions.

Single-band power in right and left-side IFG was analyzed. Pre-processed raw time-series form *Ci* was truncated from 1 s before to 1 s after the visual cue and calculated by the Short-time Fourier transform in each 5 ms window. Power in different frequencies (theta: 4–8 Hz; beta: 12–40 Hz; HG: 70–200 Hz) was filtered and Z-normalized by the same frequency power at rest (1 sec before the visual cue). The peak value in each 5 ms window after the visual cue was chosen. Cross-patient analysis in different frequency bands was compared between right and left IFG.

Age-related analysis was computed using the *Ci* signal in two parts: the power ratio in different frequency bands between Go/Correct and No-Go/Correct, and PAC. The peak values in each 5 ms window after the visual cue were chosen from the normalized power filtered in different frequency bands (theta: 4–8 Hz; beta: 12–40 Hz; HG: 70–200 Hz). The power ratio of each band between No-Go/Correct and Go/Correct was calculated in each patient by the following equation Equation (1):


(1) $$\begin{array}{r} power\;ratio\;=\;\frac{{No\;-\;Go/Correct}_{ave}}{{Go/Correct}_{ave}} \end{array}$$

where *No-Go/Correct*_*ave*_ and *Go/Correct*_*ave*_ denote the average power of No-Go/Correct trials and Go/Correct in the 1 s reaction period after the visual cue.

Moreover, PAC was computed by modulation index to determine if there was any change over IFG during Go and No-Go epochs. (23) The PAC value, *z*(*t*), was is given by Equation (2).


(2) $$\begin{array}{r} z\left(t\right)\;=\;A_{High}\left(t\right)e^{i\phi_{Low}\left(t\right)} \end{array}$$

where*A*_*High*_(*t*) is the normalized high frequency (40–200 Hz) envelope amplitude in the time series; and *iϕ*_*Low*_(*t*) is the low-frequency (2–20 Hz; stepped every 3 Hz) phase in the time series. The *z*(*t*) in 1 s period after visual was then averaged to calculate the ratio between No-Go/Correct and Go/Correct in each different-age patient.

### Statistical Analysis

The peak values in each bandpass power between Go and No-Go conditions were illustrated by mean ± 95% CI and *p*-values were calculated by unpaired *t*-test (*p* = 0.05). The Tukey–Kramer test was used to correct multiple comparisons for the unequal sizes between the Go and No-Go conditions. To test for the statistical significance of PAC between motor and IFG areas, a standard permutation test was applied by conducting 1,000 shuffles of each bin of low-frequency phase and high-frequency amplitude. This method generated a null distribution of modulating index and the 95th percentile CIs. Any real-modulating index values calculated between each bin of frequency and amplitude from *Ci* revealing greater than null distribution at an alpha level of 0.05 were considered statistically significant. The significance of PAC values of each Go/Correct and No-Go/Correct condition was calculated by a permutation test (significance value = 0.05) (25), and non-significant effects were zero-valued. The results were depicted as the ratio of the average between Go/Correct and No-Go/Correct conditions.

## Results

### Activation of High-Gamma Band Filtered Signal

**Figure 3:**
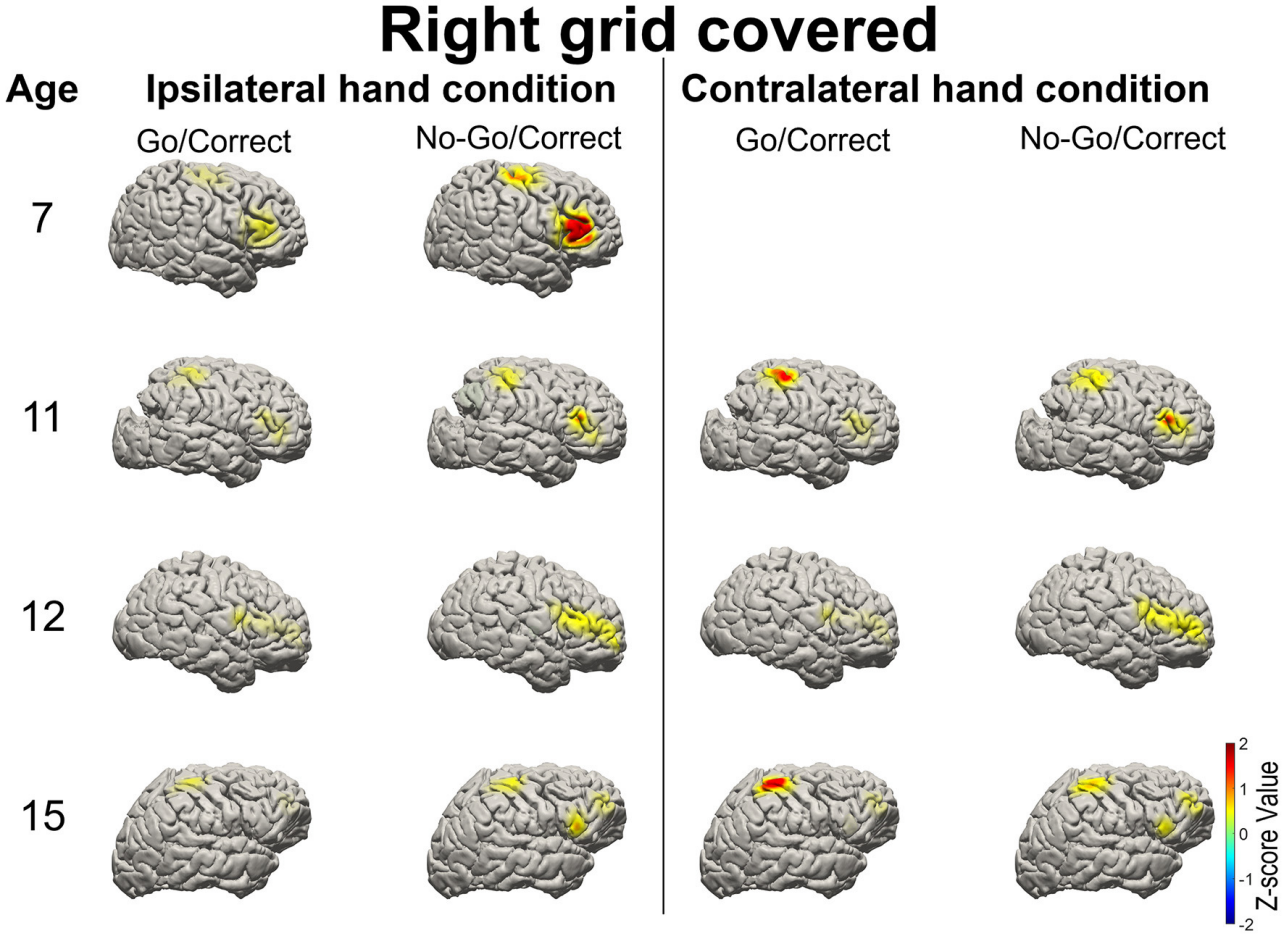
Illustration of the mean high-gamma (HG) activation for the patients aged 7, 11, 12, and 15 years with right side grid coverage. Activation of the right IFG in No-Go/Correct trials is greater than that in Go/Correct trials. HG activation over the sensorimotor cortex was observed in Go/Correct trials when the contralateral hand was used (in this case the left hand).

**Figure 4:**
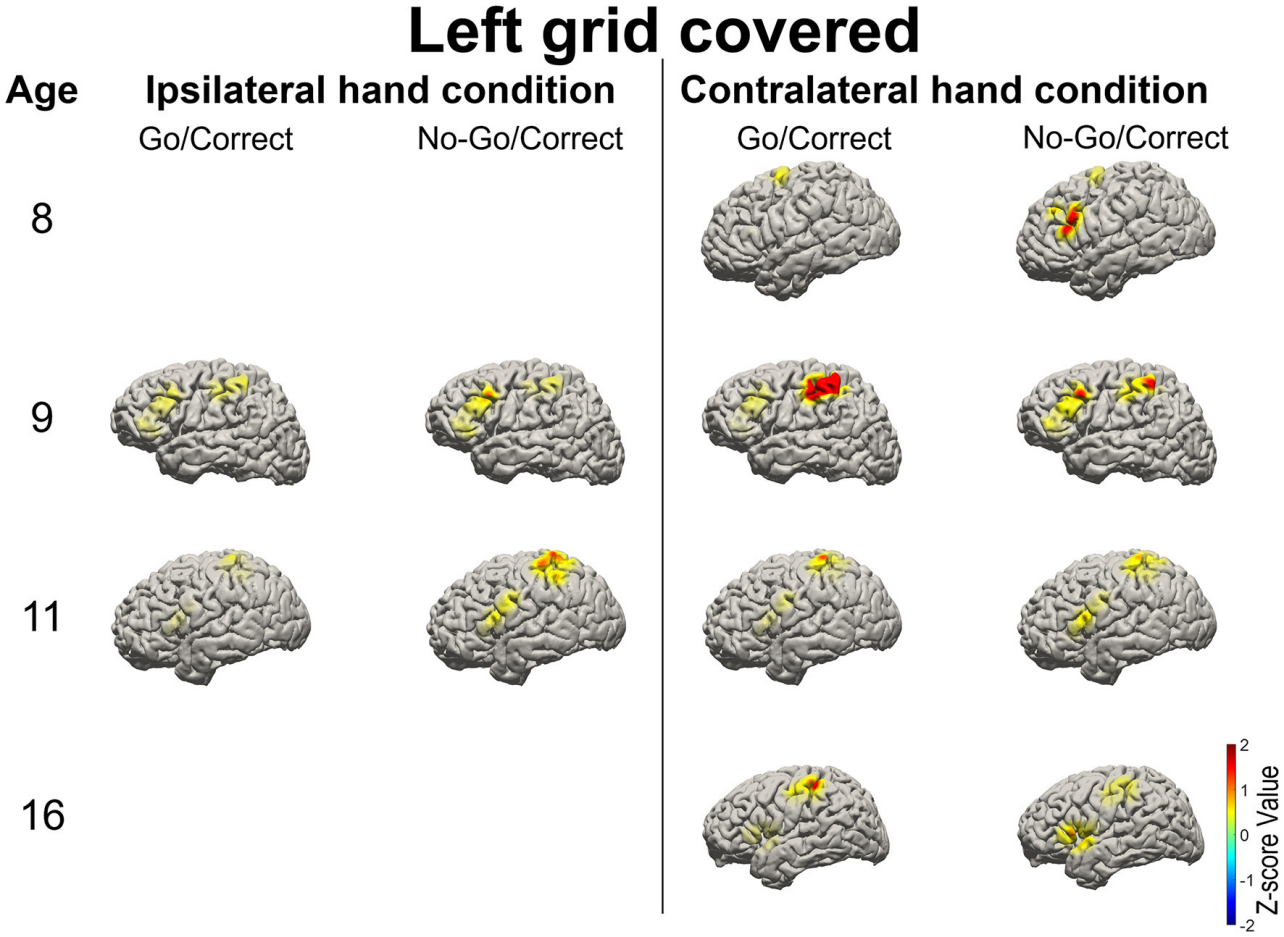
Illustration of the mean high-gamma (HG) activation for the patients aged 8, 9, 11, and 16 years with left-side grid coverage. Activation of the left IFG in No-Go/Correct trials is higher than that in Go/Correct trials. HG activation over the sensorimotor cortex was observed in Go/Correct trials when the contralateral hand was used (in this case the right hand).

High gamma-filtered ECoG power localizes movements from different body parts (35) and fine finger movements (36) across the primary motor cortex. Based on the previous findings, HG power was used to evaluate brain responses over the primary motor cortex and IFG in Go and No-Go conditions (15). For the patients with right-sided grid coverage, activated HG signals over the motor cortex were noted when the contralateral left hand was used in Go conditions (with the exception of one 12-year-old patient with no motor cortex coverage), but no activation when the ipsilateral right hand was used. Independent of which hand was used, right IFG in the No-Go condition exhibited greater HG activation relative to the Go condition (Figure 3). Similar high-frequency response profiles in the left IFG were also observed in the patients with left hemisphere grids. HG power over the motor cortex was activated when the contralateral hand was used, as shown in Figure 4.

### Power Spectrum Over Motor Cortex and IFG

**Figure 5:**
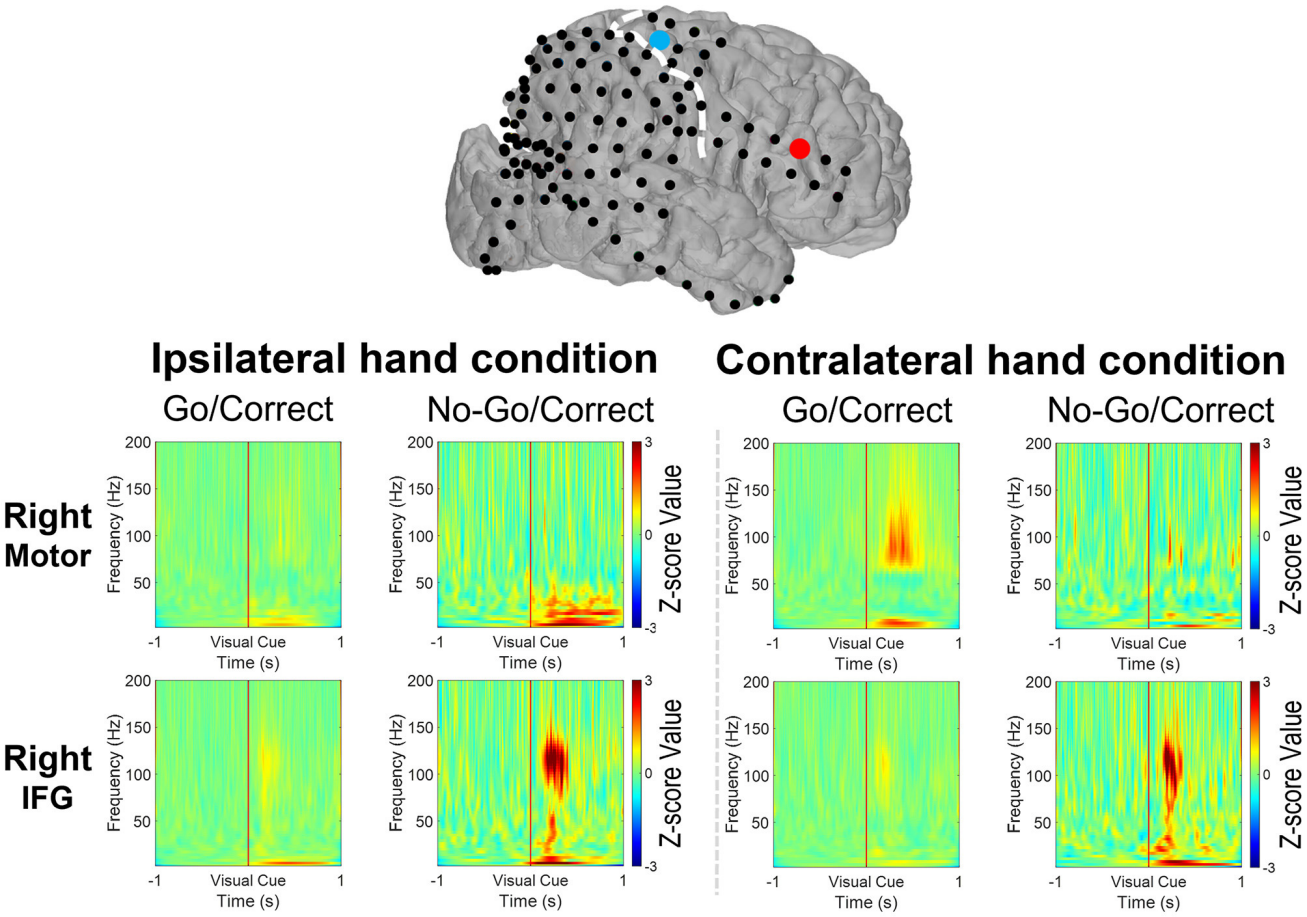
The broadband spectral changes revealed the power change over the motor cortex (the blue dot) and the IFG (the right dot) from 1-second before to 1-second after the visual cues (Go or No-Go) by the 11-year-old with a right-side grid implanted. When the left hand was used, the power within the high-gamma band (HG, 70–200 Hz) increased with the decreased beta band (12–40 Hz) over the motor cortex after visual cues in the Go/Correct trials. Increased HG, beta, and theta (4–8 Hz) power was noted when both the right and left hands were used in No-Go/Correct trials.

**Figure 6:**
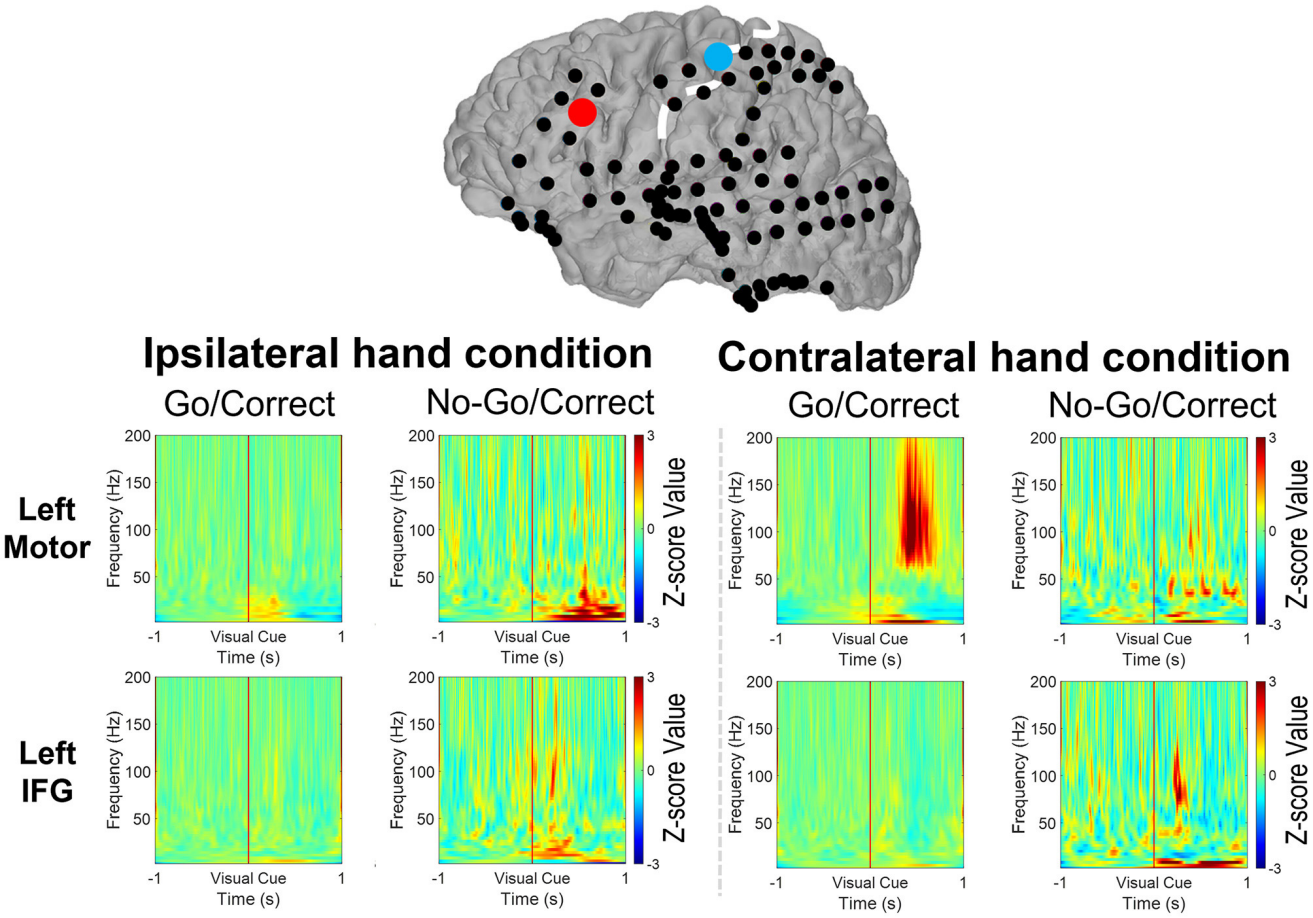
The spectrum revealed the power change over the motor cortex (the blue dot) and the IFG (the right dot) from 1 s before to 1 s after the visual cues (Go or No-Go) by a 9-year-old boy with a left-side grid implanted. When the right hand was used, the power of the high-gamma band (HG, 70–200 Hz) increased with a decreased beta band (12–40 Hz) over the motor cortex after visual cues in Go/Correct trials. Increased HG and theta (4–8 Hz) power were noted when both the right and left hands were used in No-Go/Correct trials.

**Figure 7:**
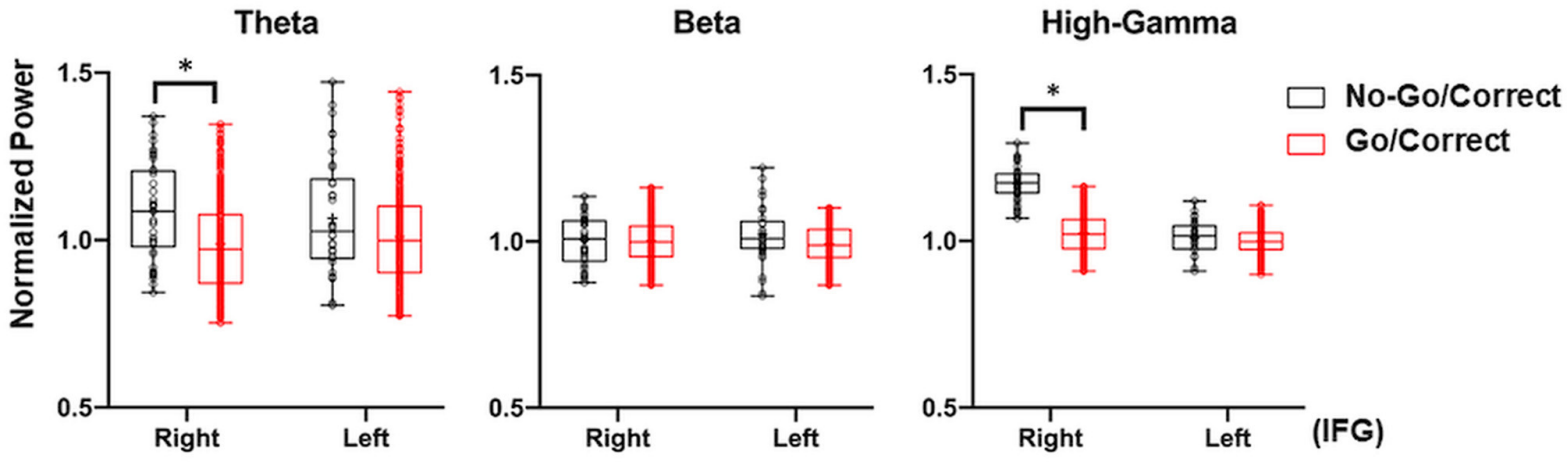
Illustration of the right and left IFG with cross-patient power analysis in Go/Correct and No-Go/Correct conditions. The normalized power in theta (4–8 Hz), beta (12–40 Hz), and high-gamma (HG, 80–200 Hz) were calculated by mean value, interquartile range (box plot), and maximal/minimal (error bar). In No-Go/Correct conditions, the power of theta, beta, and HG were higher than those in Go/Correct condition, with statistical significance in theta and HG power over the right IFG (marked by an asterisk, *p* < 0.05, corrected by the Tukey–Kramer Test).

The broadband cortical spectrum (2–200 Hz) was calculated *via* the wavelet transform from the representative electrodes of *Cm* and *Ci*. For the cortical signals from the right *Cm*, increased high-frequency power (70–200 Hz) and decreased low-frequency power (20–40 Hz) were noted after the visual cue, while the contralateral hand was used in Go/Correct conditions. This power change over the motor cortex was consistent with that of a previously published report (37). For the signal over the right *Ci*, the activated power over HG (70–150 Hz), beta (12–40 Hz), and theta (4–8 Hz) were observed when either the right or left hand was used in No-Go/Correct conditions as shown in Figure 5. Moreover, the same range of power activation in No-Go/Correct conditions was also noted over the electrode of the left *Ci*, left IFG region (Figure 6). In the cross-patient power analysis, the mean values of HG, beta, and theta power in the No-Go/Correct condition over *Ci* were higher than those in the Go/Correct conditions over both sides of the IFG, but statistical significance was only noted in the HG and theta power over the right IFG (*p* < 0.05, with corrected by Tukey-Kramer test) as shown in Figure 7.

### Age-Related ECoG Power and PAC Analysis

**Figure 8:**
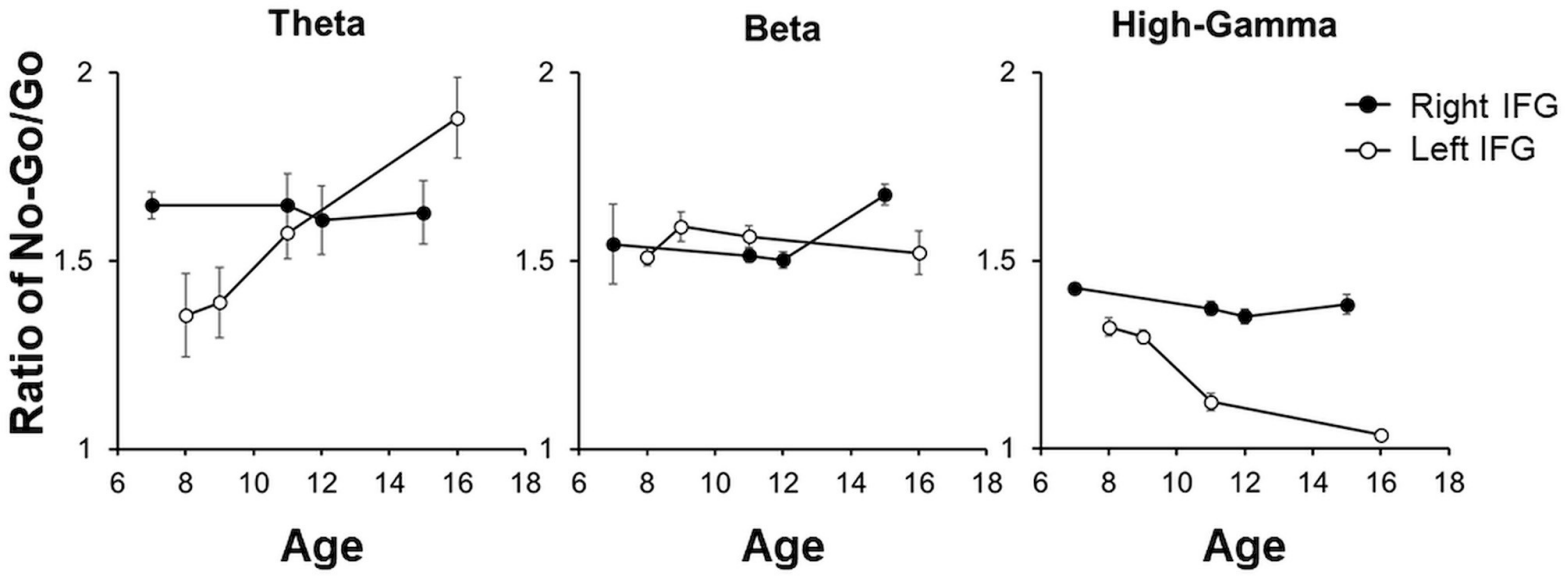
The power ratios between No-Go/Correct and Go/Correct in theta, beta, and HG were illustrated by mean ± 95% CI in each patient. For the patients with the right IFG coverage, there was no obvious change by age. For the left IFG, there was a decreasing trend in HG by age as well as an increasing trend in theta, but no obvious pattern was noted in the ratio of beta.

In each patient, the theta, beta, and HG band power ratios over the right IFG between No-Go/Correct and Go/Correct conditions were calculated. The bandpass powers among different frequency in the No-Go/Correct conditions were higher than those in the Go/Correct conditions, which mean the ratios were over 1. For the patients with grid-covered right IFG, the ratio of theta, beta, and HG frequency bands showed obvious change by age. However, with grid-covered left IFG, there was a decreasing trend of power ratio in HG by ages, and also an increasing trend in theta, but no age-related pattern was observed in the power ratio of the beta frequency band as shown in Figure 8.

**Figure 9:**
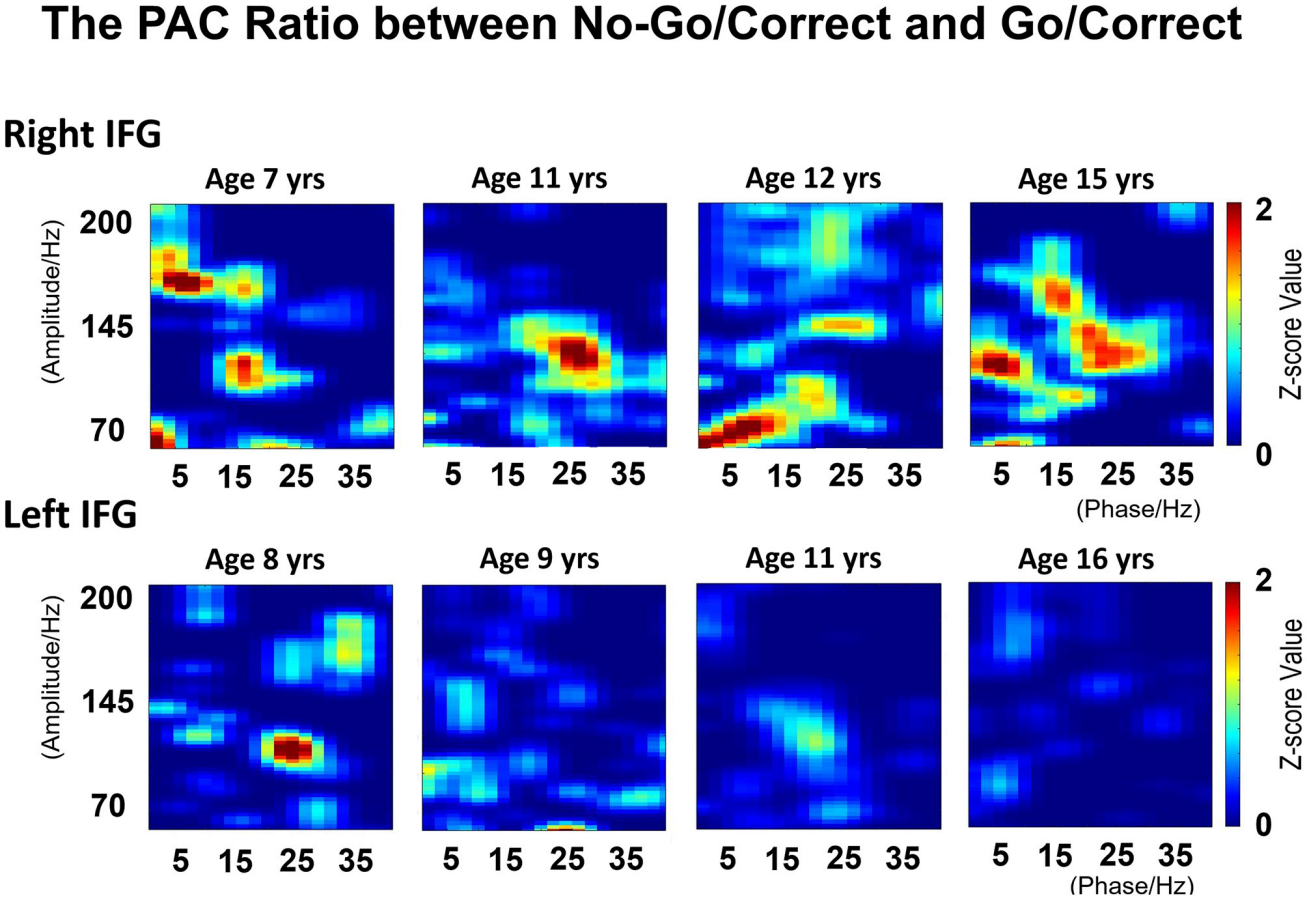
The phase-amplitude coupling (PAC) values were calculated by modulating the index between low-frequency phase (2–40 Hz) and high frequency amplitude (70–200 Hz) in Go/Correct and No-Go/Correct conditions. The illustration revealed the calculated ratio between Go/Correct and No-Go/Correct conditions in right and left-side IFGs. For the right IFG, the PAC value in No-Go/Correct conditions is higher than that in Go/Correct conditions, which means the ratio is over 1, and consistent between the low-frequency phase (5–25 Hz) and high frequency amplitude (100–150 Hz). For left IFG, a similar pattern was observed in younger patients, but the ratio decreased with age.

The PAC values over IFG (*Ci*) were computed separately for the Go/Correct and No-Go/Correct conditions. Most of the PAC values after the permutation test were non-significant (i.e., set to zero). For the right IFG, the PAC value in the No-Go/Correct conditions was higher than that in the Go/Correct conditions. Although a similar pattern was observed in the younger patients for left IFG, the ratio progressively decreased with age as shown in Figure 9.

## Discussion

In this study, we investigated movement inhibition in the eight pediatric patients who received transient subdural grid implantation for localizing epileptic foci. Our observations highlight two things. First, HG activation in No-Go conditions was higher than that in the Go condition over both the left and right side IFGs. The PAC values in the No-Go condition, calculated between low-frequency (2–40 Hz) phase and high-frequency amplitude (70–200 Hz) from IFG, were higher than those in Go conditions, and we also observed in both left and right cortices. This indicates that activation of IFG in the No-Go condition may modulate the cortical activities to inhibit movements. Second, in the left IFG, the HG and PAC ratio decreased, while the theta ratio increased, with increasing age. In the previous literature, maturational patterns of EEG activity in the resting state revealed that theta-band power decreased while the alpha-band power increased with age (38). The theta-band activity was replaced by the alpha-band first in the occipital regions and progressed later to frontal regions by ages (39). Many studies suggested changes in the white matter (WM) volume was thought to reflect the process of increased myelination, whereas myelination increases the speed of nerve impulse propagation across the brain's region-specific neurocircuitry, especially in the prefrontal cortex, up until 24 years old (40). Thus, growth-related changes in WM might lead decreasing in resting-state theta-band activity with development. In this study, we used the baseline-normalized event-related spectral perturbation (ERSP) (41) to determine the activation of ECoG data under the No-Go trial. There was an increasing power ratio of theta oscillation with stronger theta activity in No-Go trials as lower resting-state theta activity (baseline) by age. The observed age-dependent relationship between theta activity and inhibitory control at the neurophysiological and behavioral level may relate to biophysical properties of theta oscillations and their role in coordinating information processing in a network in the maturation process (42, 43). Critically, relative to adult populations, our results support the hypothesis that the center of motor inhibition in the pediatric populations is not limited to the right IFG, but rather is a product of bilateral contributions from the IFG (20).

The process of motor inhibition is generally assumed to be a multiple step, functional process, mapping onto multiple regions of the cortex (7). In an fMRI study, 26 healthy volunteers (mean age: 23.4 years old) were asked to continuously tap a button with their right index finger, stopping the movement following occasional visual cues, in order to compare the brain activation between the voluntary and forced inhibition of ongoing actions. The results revealed that during the period of inhibition there were greater activations over the SMA, middle cingulate cortex, bilateral insula, and inferior parietal cortex in addition to the right IFG (44). In another fMRI study of 26 healthy subjects, BOLD signals were measured in a Go/No-Go task. Greater activation over the right IFG and pre-SMA regions were noted in No-Go conditions than in Go conditions (45). A 15-participant study (mean age = 27.5 years old) compared different degrees of difficulty of the stop signal task and found that the right IFG and adjacent anterior insula had more activation during more difficult tasks (46). Additional insight regarding the role of the left IFG in Go/No-Go task execution was demonstrated in a 22-participant fMRI study. The study included two variants of the Go/No-Go ratio: a high frequency of Go cues (Go: No-Go = 3: 1), and a high frequency of No-Go cues (Go: No-Go = 1: 3). The results revealed that the left IFG and a dorsal portion of the pre-SMA were more reactive to No-Go cues compared with Go cues, whether the frequency of No-Go cues was high or low (47). Together, these studies indicate that the right IFG participates in a network that orchestrates the process of motor inhibition. The findings of this study support the conclusion that in addition to the right IFG, which is well-established as being involved in motor inhibition in adults, the left IFG also plays a role in motor inhibition within the maturing brain (20).

Considering the whole neural network for movement inhibition, the change of neural activations over extended regions of the cortex, such as motor, premotor, and pre-SMA regions, would also be observed between Go and No-Go conditions. In a stop-event-related study of 12 adult epileptic patients, event-related spectral power was measured by intracranial ECoG to identity the movement-related spectral change over the sensorimotor cortex. The early increased *mu* band (10–20 Hz) reflected a transient state of motor inhibition over the precentral gyri (48). In another Go/No-Go task, the middle frontal gyrus (MFG) demonstrated transiently increased HG power during stop signals, and the increased HG over MFG was stronger for unsuccessful stop conditions compared to successful stop conditions, which implied the role of MFG in behavioral monitoring (49). The processing of motor inhibition not only included the frontal region, but also the pre-SMA, orbitofrontal cortex, dorsolateral pre-frontal cortex, and basal ganglia (3–6).

Modulating effects between different brain regions, including the IFG, anterior insula, pre-SMA, and sensorimotor cortex, with interaction with basal ganglia, were demonstrated in a neural network study of movement inhibition (47). The movement context was modulated by the neural activity of the basal ganglia, anterior cingulate cortex, and frontal cortex (50, 51). In an fMRI study, response-related amplitudes were calculated *via* logistic regression analysis. Covariance was applied to evaluate the coupling effects between two regions. The results revealed the coupling between the fronto-parietal regions and right IFG increased in successful stop signal tasks compared with that of unsuccessful stop signal tasks, suggesting that the right IFG had more neural interaction during movement inhibition (52). The role of the left IFG in movement inhibition was also evaluated by correlation in another fMRI study by a Go/No-Go task. In No-Go trials, the left IFG revealed positive connectivity with the dorsal portion of the pre-SMA, but negative connectivity with regions responding to Go cues (left sensorimotor cortex) (47). In a clinical case report, the fMRI data analysis in the Go/No-Go task revealed left IFG compensated the original right IFG function after brain injury, which may be the reactivation of the original left IFG function (53). These findings are congruent with the results of our study, which revealed that both the right and left IFG serve to modulate focal activity with the motor cortex during No-Go conditions.

Age-related neural development has been widely discussed, including changes in cortical thickness (16), cortical structure (54), the number of synapse formations (55), and functional activity (54). For example, visual acuity has been correlated to the structure and thickness of the visual cortex (16). Most neural development, such as the sensorimotor cortex, matures symmetrically over both sides (56). However, the language function lateralizes to the dominant cortex with age (57). Utilizing structural MRI, lateralization was strongly correlated with volume and thickness over the left IFG (58). For the function of motor inhibition, the presented studies show that right IFG plays an important role (2, 7, 8, 15), but some reports have revealed equal contribution by bilateral IFG (20). In an MRI study, decreasing WM tracts over right IFG was observed in the patients with Attention Deficit Hyperactivity Disorder, compared with the normal population. For the motor inhibition, the results identified the pathogenesis of WM tracts over IFG potentially related to deficient inhibitory control (59). Our study indicated that both IFG played a role in motor inhibition in the pediatric population, but with age, lateralization to the right IFG becomes dominant.

There were some limitations in this study. The brain activity recorded by the ECoG signal was limited by the location of grids and strips, and coverage of the regions of interest was determined entirely by the clinical need. In the future, more cases are needed to investigate the correlation between age and motor inhibition, which may reveal the developmental pattern from bilateral IFG involvement in children to solely right IFG involvement in adults.

## Conclusion

In our pediatric patients, both the right and left IFG had roles in motor inhibition. The power ratio between the No-Go and Go conditions revealed age-related lateralization from the bilateral-to-right side IFG. The PAC modulation over IFG was more synchronized in No-Go trials than in Go trials. The correlation of age computed by the PAC ratio between two conditions further supported age-related right side IFG lateralization.

## Data Availability Statement

The datasets generated for this study are available on request to the corresponding author.

## Ethics Statement

The studies involving human participants were reviewed and approved by Seattle Children's Hospital Institutional Review Board. Written informed consent to participate in this study was provided by the participants' legal guardian/next of kin.

## Author Contributions

C-HK, KCa, JW, PR, KW, and JO designed the study. C-HK, KCa, JW, KCo, and JO participated in data collection. EN and JO provided the data resource. C-HK, KCa, B-WC, S-HY, Y-CL, and Y-YC analyzed the data. C-HK, KCa, KCo, Y-YC, Y-CL, KW, and JO wrote and reviewed the manuscript. All the authors reviewed and approved the final version of the manuscript.

## Funding

We are grateful for support from the Ministry of Science and Technology of Taiwan under Contract numbers of MOST 110-2321-B-010-006, 109-2811-E-010−502, 109-2221-E-010-004-MY2, and 108-2321-B-010-008-MY2. We also are grateful for support from the Higher Education Sprout Project of the National Chiao Tung University and Headquarters of University Advancement at the National Cheng Kung University, Ministry of Education (MOE), Taiwan.

## Conflict of Interest

The authors declare that the research was conducted in the absence of any commercial or financial relationships that could be construed as a potential conflict of interest.

## Publisher's Note

All claims expressed in this article are solely those of the authors and do not necessarily represent those of their affiliated organizations, or those of the publisher, the editors and the reviewers. Any product that may be evaluated in this article, or claim that may be made by its manufacturer, is not guaranteed or endorsed by the publisher.
